# Single- and Multicomponent Siloxane Adsorption in Al-MCM-41 (Al = 0.0, 1.3, and 4.4)

**DOI:** 10.1021/acsomega.5c03379

**Published:** 2025-09-08

**Authors:** Camila M. A. C. Alves, Júlia F. Alves, Raimundo C. Rabelo-Neto, Luiz S. C. Júnior, Araceli Fuerte, Paloma Ferreira-Aparicio, Rita X. Valenzuela, Rinaldo S. Araújo, Mona Lisa M. Oliveira

**Affiliations:** † Universidade Estadual do Ceará (UECE), Fortaleza, Ceará 60740-000, Brazil; ‡ Instituto Federal de Educação, Ciência e Tecnologia do Ceará, Campus de Fortaleza (IFCE), Fortaleza, Ceará 60040-531, Brazil; § Instituto Nacional de Tecnologia (INT), Rio de Janeiro, Rio de Janeiro 20081-312, Brazil; ∥ Centro de Pesquisas, Desenvolvimento e Inovação Leopoldo A. M. de Mello (Cenpes), Petróleo Brasileiro S.A. (PETROBRAS), Rio de Janeiro, Rio de Janeiro 21941-915, Brazil; ⊥ Centro de Investigaciones Energéticas, Medioambientales y Tecnológicas (CIEMAT), Madrid 28040, Spain

## Abstract

Adsorption studies in the liquid phase at 25 °C of siloxanes L5 (linear) and D4, D5, and D6 (cyclic) on silica MCM-41 (pure or Al-doped) were performed, including evaluations of siloxane removal in single- and multicomponent forms. The materials were synthesized by the sol–gel method and characterized by techniques such as XRD, SEM-EDS, ICP-OES, FT-IR, N_2_ adsorption–desorption at 77 K, NH_3_-TPD, and NMR. The characterization results confirmed the chemical and textural properties typical of mesoporous materials. The order of adsorption capacity was Al-MCM-41(10) ≫ Al-MCM-41(30) ≥ MCM-41 for all siloxanes (L5, D4, D5, and D6) in the different scenarios (single, binary, and ternary components), which was influenced by chemical structure, molecular geometry, and adsorbent acidity. The Al-MCM-41(10) adsorption capacities were 0.479, 0.097, 0.055, and 0.045 mmol g^–1^ for L5, D4, D5, and D6, respectively. The Al-MCM-41(10) adsorption kinetics demonstrated that the siloxane adsorption process followed pseudo-first-order kinetics across all scenarios. Recovery tests showed appreciable adsorptive capacities (100–76%) of D4 on Al-MCM-41(10) at 25 °C for three consecutive uses. The results showed promising characteristics for the physical adsorption of linear and cyclic siloxanes on easy synthesis materials under less expensive operational conditions.

## Graphical abstract

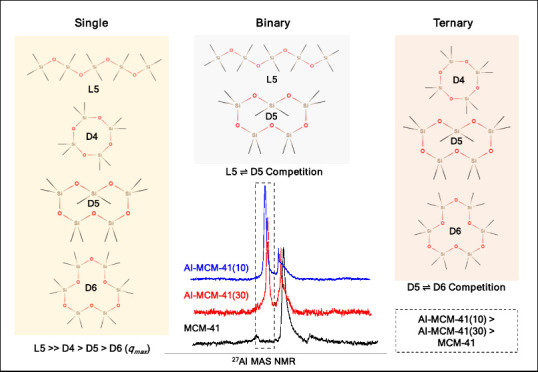

## Introduction

Siloxanes are organosilicon compounds characterized by silicon–oxygen (Si–O) and methyl (−CH_3_) groups bonded to the silicon atoms.^1^ Compounds with molecular weights below 500 g mol^–1^ are named volatile methyl siloxanes (VMS) and, depending on their structure, can be classified into linear (L) and cyclic (D) forms.^2^ Octamethylcyclotetrasiloxane (D4), decamethylcyclopentasiloxane (D5), and dodecamethylcyclohexasiloxane (D6) are the siloxanes most commonly found in environmental matrices and biogas.^3,4^ The dodecamethylpentasiloxane (L5) is found in lower concentrations; however, further studies are needed due to its unknown potential for long-range transport and bioaccumulation.^5^ Siloxanes possess distinct physicochemical properties, including low flammability, low surface tension, and high thermal stability, and raise concerns about their frequent use in industrial products.^6^ In addition, the VMS are extremely volatile, with a relatively long half-life in air (6–11 days) and low solubility in water.^6,7^

The indiscriminate use of VMS can lead to significant environmental contamination in the aqueous phase, as the widespread dispersion of these compounds results in their introduction into wastewater treatment plants.^8^ Their low biodegradability and strong affinity for dissolved and particulate matter also contribute to their transfer from wastewater to sludge in treatment plants.^3,5^ Several studies highlight the importance of monitoring siloxanes in industrial wastewater, municipal sewage, and natural water sources to prevent risks to human health and protect the environment. From an energy perspective, siloxanes play a prominent role as common pollutants in biogas used as a biofuel. To fully harness the energy potential of methane (CH_4_), this raw material must undergo extensive purification processes to produce biomethane.^9,10^ During the combustion of biomethane contaminated with siloxanes, the formation of potentially corrosive, toxic, and flammable products and silicates can lead to wear or deposits on engine components, reducing thermal and electrical conductivity.^5,11^

The literature addresses several technologies for siloxane removal, where adsorption appears as one of the most efficient processes.^12^ Adsorption is the process by which molecules of a substance adhere to the surface of a solid, enabling the separation of mixture components.^13^ One of the materials that can be used is zeolites, of which there are a variety, such as zeolite Socony Mobil-5 (ZSM-5), mordenite (MOR) zeolites, ultrastable Y (USY) zeolite, beta zeolites (BEA), and others. Despite their high BET surface area and distinct structures with abundant acid sites, the zeolite crystallinity can hinder the diffusion of large molecules into the micropores.^14^ Due to this, mesoporous materials based on silica (M4S1 family), such as MCM-41, constitute interesting alternatives for large molecule separation.^15^ Its hexagonal, uniform, and one-dimensional mesopore structure (2–10 nm) allows for a broad range of applications in the fields of adsorption^16^ and catalysis.^17^ Additionally, their high surface area, good chemical and thermal stability, and the possibility of incorporating aluminum into the mesoporous matrix confer improvements such as acidity, causing better adsorption.^13,18^

Some studies have already investigated the mesoporous materials used for siloxane removal in the gas^19^ and liquid^20^ phases. However, in addition to studying the adsorbate as isolated components, it is essential to assess the adsorptive competition between siloxanes so that the adsorption behavior is analyzed in a manner that more accurately reflects real contamination scenarios, mainly in the liquid or aqueous phase, since few studies have investigated this behavior. Vega-Santander et al.^21^ used variants of UTD-1 pure silica zeolites with a DON-type framework for monomethylsilanetriol (MMST), dimethylsilanediol (DMSD), and trimethylsilanol (TMS) removal in single and multicomponent modes. A recent study by this research group evaluated the use of MCM-41 for L5 and D5 removal in the liquid phase in single and binary form, as well as computational studies. DFT simulations to calculate the adsorption energies showed −0.76 eV for the MCM-L5 system, significantly higher than the −0.50 eV found for MCM-D5, which can be related to the structural difference of both siloxanes. This study corroborated the experimental evaluation, where it was observed that D5 presented a lower adsorption capacity (0.019 and 0.032 mmol g^–1^ for single and binary components, respectively) compared to L5 (0.058 and 0.054 mmol g^–1^ for the single and binary components, respectively).^22^

In order to functionalize the material to obtain better adsorption capacities, as well as to evaluate the competitiveness between adsorbates, the present work aims to carry out equilibrium adsorption studies in the liquid phase (isooctane) at room temperature (25 °C) of siloxanes L5 (linear) and D4, D5, and D6 (cyclic) in single, binary, and ternary components, using three adsorbent mesoporous materials based on silica Al-MCM-41 (Al = 0.0, 1.3 and 4.4). Furthermore, the most efficient material was used to perform adsorption kinetics and adsorbent regeneration studies. This research is environmentally, technologically, and methodologically significant. Environmentally, it helps reduce pollutants in wastewater, which encourages cleaner industrial practices. Technologically, it improves energy efficiency, especially in processes involving biofuels. Additionally, it advances the methodological development of functionalized MCM-41 matrices, with aluminum or other metals, for siloxane adsorption studies (mainly in the liquid phase), also aiming to optimize resources.

## Experimental Section

### Reagents

The siloxane standards (Sao Paulo, Brazil, Sigma-Aldrich) used in the study were dodecamethylpentasiloxane (L5, 384.8 g mol^–1^), octamethylcyclotetrasiloxane (D4, 296.6 g mol^–1^), decamethylcyclopentasiloxane (D5, 370.8 g mol^–1^), and dodecamethylcyclohexasiloxane (D6, 444.9 g mol^–1^); their structures can be observed in Figure 1. Tetraethyl orthosilicate (TEOS, Saint Louis, United States, Sigma-Aldrich) and aluminum triisopropoxide (AlTIPO, Sao Paulo, Brazil, Thermo Scientific Chemicals) were used as sources of silica and aluminum, respectively. Dodecylamine (Sao Paulo, Brazil, Sigma-Aldrich) was used as a template for the synthesis of mesoporous materials, and HCl (Sao Paulo, Brazil, Sigma-Aldrich) was used to acidify the solution. In contrast, NH_4_OH (Rio de Janeiro, Brazil; Isofar) was used in the alkaline condensation step. The solvent used for synthesis was ethanol 99.9% HPLC grade (Darmstadt, Germany, Merck). The organic siloxane solutions (single, binary, and ternary) were prepared with isooctane HPLC grade (Darmstadt, Germany, Merck). For the solution siloxane analysis by gas chromatography with flame ionization detector (GC-FID), helium was employed as a carrier gas, and other gases, including hydrogen and nitrogen, as well as synthetic air, were also used. All gases used are of analytical grade (Rio de Janeiro, Brazil, White Martins). No unexpected or unusually high safety hazards were encountered.

**Figure 1:**
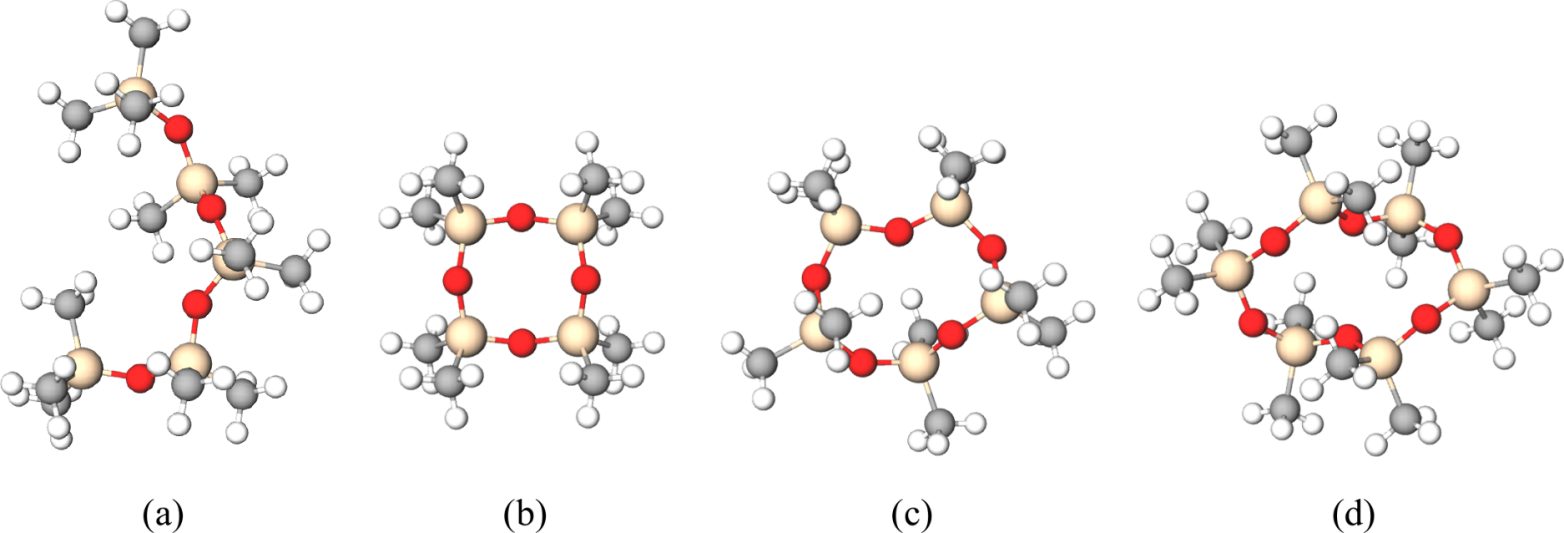
Siloxanes: (a) L5, (b) D4, (c) D5, and (d) D6. Silicon atoms are represented in beige, oxygen in red, carbon in gray, and hydrogen in white— Source: Molview (https://molview.org).

### Synthesis of Materials

Three mesoporous materials were prepared. The first was developed without the addition of Al in the silica structure (MCM-41), and the other two samples were obtained in 30 and 10 Si/Al molar ratios, which were designated as Al-MCM-41(30) and Al-MCM-41(10), respectively. The synthesis followed the sol–gel method described in previous studies.^22^ Silicon and aluminum alkoxides (TEOS and AlTIPO) underwent acid hydrolysis in an ethanolic solution containing dodecylamine at pH 2 for 75 min, with magnetic stirring at 25 °C, followed by an alkalization step to pH 10 with aqueous NH_4_OH. Each suspension was aged in an ammoniacal medium for 15 h, and the three precipitates were washed in a 1:1 ethanol/0.5 M HCl solution for 24 h. The solids were washed with deionized water, filtered, and dried at 105 °C for 12 h. Final calcinations were carried out in a muffle furnace, heating from 100 to 550 °C at a rate of 10 °C min^–1^ over 3 h. The synthesis yield was defined considering the weight of each final product—MCM-41, Al-MCM-41(30), and Al-MCM-41(10)—obtained experimentally, relative to the weight of the precursors used in the initial mixture. The molar compositions of the three synthesis gels can be verified in Table S1.

### Characterization

The chemical composition and morphology of material samples were performed using a field emission gun with a scanning electron microscope (FEG-SEM, ZEISS, Auriga 40) equipped with a Bruker E-Flash detector and an energy-dispersive spectroscopy (EDS) module integrated into the microscope system. The Al incorporation into the MCM-41 structure was also determined by inductively coupled plasma optical emission spectrometry (ICP-OES, Agilent Technologies 5100). For this analysis, the monitored Al wavelength was 396.2 nm, and the samples (50 mg) were previously prepared. X-ray diffraction (XRD, model 7000, Shimadzu) with a CuKα radiation source (λ = 1.5418 Å), operating at 40 kV and 30 mA, with a low-angle range (2° < 2θ < 6°), was used for crystallographic sample analysis.

A Fourier-transform infrared spectrometer (FT-IR, Spectrum 100 FT-IR, PerkinElmer) was used to collect spectra within the 450–4000 cm^–1^ range, with a resolution of 4 cm^–1^, where samples were prepared as KBr pellets.

The nuclear magnetic resonance spectra were carried out at room temperature (22 °C) following the methodology of Canhaci et al.^23^ An NMR spectrometer (Agilent DD2 400) features a 9.40 T wide-bore magnet operating at a Larmor frequency of 103.89 MHz for ^27^Al. Samples were packed into 4 mm ZrO_2_ rotors sealed with Kel-F caps and spun at an MAS rate of 10 kHz using a triple-resonance HXY probe. Acquisition parameters included a radiofrequency (RF) field strength of approximately 110 kHz, a pulse length of 0.9 μs (π/20), a recycle delay of 0.5 s, a spectral width of 75 kHz, an acquisition time of 20 ms, and 10,000 scans. For the ^29^Si CP/MAS spectra, data were collected on a Bruker NEO 500 spectrometer with an 11.75 T standard-bore magnet operating at 99.37 MHz. Powdered samples were packed into 4.0 or 7.0 mm ZrO_2_ rotors and spun at 5 kHz by using double-resonance HX probes. Spectra were recorded using a ramped cross-polarization (CP) sequence with a contact time of 5.0 ms, a recycle delay of 5.0 s, an RF field strength of 80 kHz, a spectral width of 50 kHz, an acquisition time of 20 ms, and 4096–16384 scans. High-power ^1^H decoupling (SPINAL-64, 90 kHz) was applied during the acquisition. All spectra were processed using Fourier transform with 50 Hz line broadening. Chemical shifts were referenced to TMS, with AlCl_3_.6H_2_O and kaolin serving as secondary standards for ^27^Al (0.0 ppm) and ^29^Si (−91.5 ppm), respectively.

Nitrogen adsorption–desorption isotherms were measured at 77 K to analyze the textural properties of the materials. The Brunauer–Emmett–Taylor (BET) method was used on the adsorption data within the relative pressure range of 0.05–0.30 to determine the specific surface area. Pore diameter and pore volume were estimated with the Barrett–Joyner–Halenda (BJH) method.

Surface acidity was assessed through temperature-programmed desorption of ammonia (NH_3_-TPD). Measurements were performed in a U-shaped quartz reactor with an internal diameter of 12 mm containing 0.1–0.15 g of catalyst under atmospheric pressure. Samples were initially exposed to a 4 vol % NH_3_/He gas stream at a flow rate of 30 mL min^–1^ and kept at 100 °C for 30 min. Next, the system was purged with pure helium (50 mL min^–1^) for another 30 min at the same temperature to remove physisorbed ammonia. Desorption was then carried out by heating the sample to 650 °C at a rate of 17 °C min^–1^. A Balzers-Pfeiffer mass spectrometer was used in the continuous monitoring of desorbed species.

### Adsorption Experiments

Adsorption experiments were conducted to determine the adsorption capacities of MCM-41, Al-MCM-41(30), and Al-MCM-41(10), using four siloxanes in single (L5, D4, D5, and D6), binary (L5 and D5), and ternary (D4, D5, and D6) systems. For this, the experiments were divided into 4 stages: (i) Adsorption equilibrium (single) on the three synthesized materials, (ii) adsorption equilibrium (single, binary, and ternary) on the most efficient material; (iii) adsorption kinetics (single, binary, and ternary) on the most efficient material, and (iv) regenerability studies on the most efficient material.

Equilibrium adsorption experiments were performed using 50 ± 5 mg of each adsorbent in 20 mL of isooctane containing siloxanes in single, binary, and ternary solutions. For cyclic siloxanes (D4, D5, and D6), concentrations ranged from 0.022 to 0.680 mmol L^–1^ (10–200 mg L^–1^), whereas for the linear siloxane (L5), concentrations ranged from 0.026 to 1.00 mmol L^–^^1^ (10–400 mg L^–1^). Each suspension experiment was stirred in a shaker (Marconi, MA 410) at 25 °C for 144 h to ensure equilibrium. Siloxane quantifications were performed using a gas chromatography method^24^ with flame ionization detection (GC-FID, TRACE GC, Thermo Scientific) equipped with a manual injection system (1 mL loop) and a capillary column (BP-624, Capillary GC Column, SGE) of 30 m × 0.53 mm × 3 μm. The oven temperature was programmed from 80 to 200 °C at a heating rate of 20 °C min^–1^. Calibration was performed using external standards prepared from stock solutions containing single binary or ternary siloxanes.

Kinetic adsorption experiments were conducted using 50 ± 5 mg of the most efficient adsorbent added to 20 mL of isooctane solution containing single, binary, and ternary siloxanes at a fixed initial concentration (0.5 mmol L^–1^). Aliquots were withdrawn at predetermined time intervals (0, 4, 8, 24, 48, 72, 96, 120, and 144 h), and the siloxane concentrations were quantified using the same GC-FID method. The adsorption capacity, as well as the equilibrium (Langmuir, Temkin, and Dubinin–Radushkevich), and kinetic (pseudo-first order, pseudo-second order, and intraparticle diffusion) models for siloxanes under single, binary, and ternary scenarios, were calculated using equations reported in previous studies.^22,25,26^

An adsorbent recovery/regeneration experiment was carried out according to the thermal regeneration method adapted from Yassin et al.^27^ for the adsorbent/adsorbate system that showed a greater capacity in siloxane removal by adsorption. The process was performed in consecutive contact and regeneration (calcination) steps. Siloxane was adsorbed in a medium containing 100 mL of isooctane and 250 mg of the adsorbent in each cycle. After the equilibrium time (144 h), an aliquot was removed, and the adsorption capacity was determined. Then, the adsorbent was regenerated by calcining the material in a muffle furnace at the same temperature as that used in the synthesis step. The cycles were repeated until adsorption was no longer effective.

## Results

### Characterization

Based on the initial amount of silicon or silicon/aluminum metal alkoxides added, the estimated synthesis yields were 84.6, 90.7, and 91.8% for MCM-41, Al-MCM-41(30), and Al-MCM-41(10), respectively, which makes them viable for large-scale reproduction. The results of the chemical composition analyses and the Si/Al ratio obtained by EDS and ICP-OES are shown in Table 1 below.

**Table 1:** Chemical Composition of Materials by EDS and ICP-OES

	composition (wt %)			molar ratio Si/Al		
sample	Si	O	Al	theoretical	EDS	ICP-OES
**MCM-41**	47.9^a^	52.1^a^	-	-	-	-
AL-MCM-41(30)	54.0^a^	45.1^a^	0.910^a^ (1.32)^b^	30	48.1	33.2
**AL-MCM-**41(10)	51.6^a^	45.9^a^	2.50^a^ (4.38)^b^	10	16.6	10.6

a EDS Al determination.

b ICP-OES Al determination.

The composition results show relative differences between the techniques used, which can be attributed to the adaptation made in the synthesis methodology and the differences between them. The higher percentage of Al observed by ICP-OES shows a high Al incorporation in the structure, close to the Si/Al molar ratio in the synthesis gel. On the other hand, the lower Al contents (wt %) by EDS may be related to the lower analytical sensitivity of the technique, which infers only the surface composition of the solid. Figure 2 shows the other characterization performed on the three synthesized materials.

**Figure 2:**
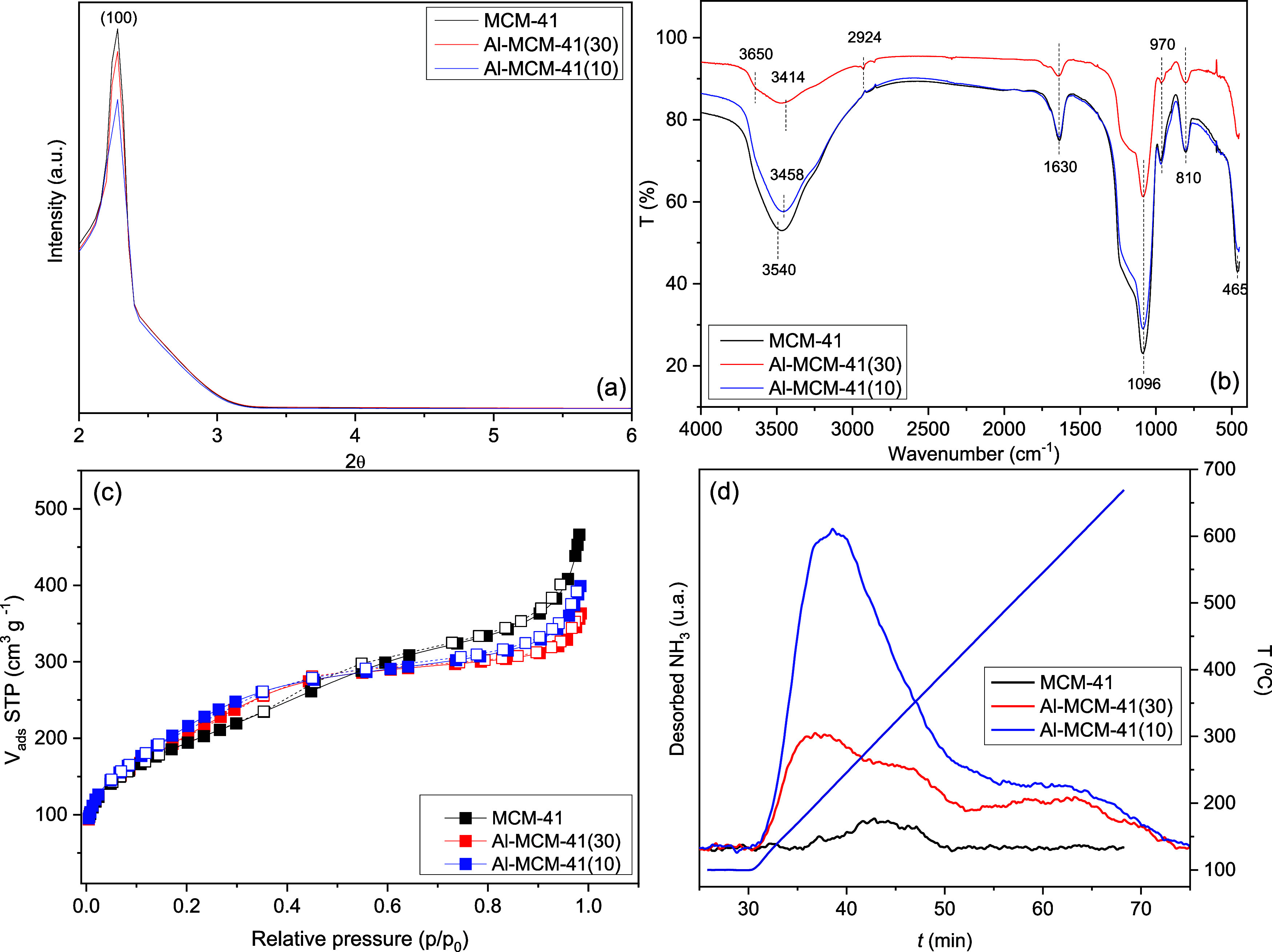
Physicochemical, spectroscopic, textural properties, and morphology of samples: (a) XRD diffractogram presented a peak at 2θ = 2.28°, corresponding to the (100) reflection plane in all materials; (b) FT-IR spectra show bands related to the symmetric and asymmetric stretching vibrations of the O–H, Si–O–Si, and Al–O–Si bonds; (c) N_2_ adsorption–desorption isotherms at 77 K indicate that the materials present type IV isotherms, typical of mesoporous materials; (d) TPD-NH_3_ showed that the MCM-41 material does not have measurable surface acidity, with an increase in acidity corresponding to the rise in aluminum content in Al-MCM-41.

The XRD diffractograms (Figure 2a) exhibit an intense peak at 2θ = 2.28° attributed to the reflection line of the (100) plane, characteristic of the MCM-41 materials with the ordered hexagonal structure.^15,28^ Other peaks common to mesoporous materials, such as the (110) and (200) planes in the 2θ range of 3.5 to 4.5°, were not observed, likely due to a short-chain surfactant and the lower synthesis temperature.^29,30^ The interplanar distance (d_100_) and the lattice parameter (α_0_) related to the hexagonal system of the matrices showed that the hexagonal structure was preserved even with Al incorporation into the MCM-41 mesoporous structure. The values can be checked in Table S2.

The FT-IR spectrum (Figure 2b) exhibits similarities among the three materials because the symmetric and asymmetric stretching vibrations of Si–O–Si and Al–O–Si bonds occur in the same spectral range.^31^ The spectra exhibited a broad band in the 4000–3000 cm^–1^ region, corresponding to stretching vibrations of adsorbed water on the structural surface. This region also includes contributions from O–H stretching vibrations of silanol groups (Si–OH) present in different configurations: isolated (∼3750 cm^–1^), internally bonded within the silica network (∼3650 cm^–1^), and hydrogen-bonded (∼3540 cm^–1^).^32^ The 1630 cm^–1^ band can be attributed to the bending vibrations of the O–H in the plane, while the broad absorption between 1250 and 1000 cm^–1^ corresponds to the Si–O–Si asymmetric stretching in the structure. Furthermore, these same bands may be related to the vibrations of symmetric and asymmetric stretching associated with Al–O–Si bonds in Al-MCM-41, and the weak band appearance at 970–800 cm^–1^ is good evidence of the Al isomorphic incorporation (Si–O–Si or Si–O–Al stretching vibration).^31–33^

The N_2_ adsorption–desorption isotherms (Figure 2c) indicate that the materials present type IV isotherms, typical of mesopore materials according to the IUPAC classification.^34^ In the type IV isotherm, the monolayer formation is observed, followed by capillary condensation and, finally, the adsorption of multilayers until inflection and saturation of the isotherm. All samples exhibited monolayer adsorption ranging from 97 to 200.9 cm^3^ g^–1^ STP at relative pressures (p/p_0_) of <0.18. The isotherms exhibited weak hysteresis loops in the *p*/*p*_0_ of 0.35–0.80 for MCM-41 and Al-MCM-41(10), suggesting uniform mesopore filling and the absence of additional crystalline phases or significant pore blockage, thereby facilitating N_2_ diffusion.^35^ In contrast, the Al-MCM-41(30) material presented more pronounced hysteresis at the same relative pressures. The samples exhibited the following structural and textural properties for MCM-41, Al-MCM-41(30), and Al-MCM-41(10), respectively: BET surface areas of 694, 769, and 812 m^2^ g^–1^, mean pore diameters of 4.04, 2.87, and 2.96 nm, and pore volumes of 0.701, 0.551, and 0.601 cm^3^ g^–1^. The structural parameter values in all samples confirm the mesoporous nature of the solids obtained, although the estimated BET surface areas are relatively small for this type of material. The values obtained can be considered close to those found in the literature.^22,29,36,37^ The aluminum incorporation in the mesoporous structure MCM-41 determined moderate increases in the surface area for the synthesized aluminosilicates and a reduction in the diameter and volume pore values, consistent with the findings of Fang et al.^38^

TPD-NH_3_ analysis (Figure 2d) shows that the MCM-41 material exhibits no measurable surface acidity. Comparatively, the Al-MCM-41 samples mostly displayed moderately strong sites between 250 and 400 °C, with peaks at 260 °C for the Al-MCM-41(30) and 300 °C for the Al-MCM-41(10) material. Furthermore, the more acidic material shows strong acidic sites at temperatures ranging from 450 to 600 °C, corresponding to NH_3_ desorption times between 55 and 70 min. The acid site density values for the MCM-41, Al-MCM-41(30), and Al-MCM-41(10) materials were 7, 76, and 149 μmol of NH_3_ g^–1^, respectively. Despite the low values compared to the original methodology, the values found are within the same range as those reported by Iliopoulou et al.,^39^ Zhao et al.,^40^ and Pham et al.^41^ Finally, the NMR spectra and SEM images are presented in Figure 3.

**Figure 3:**
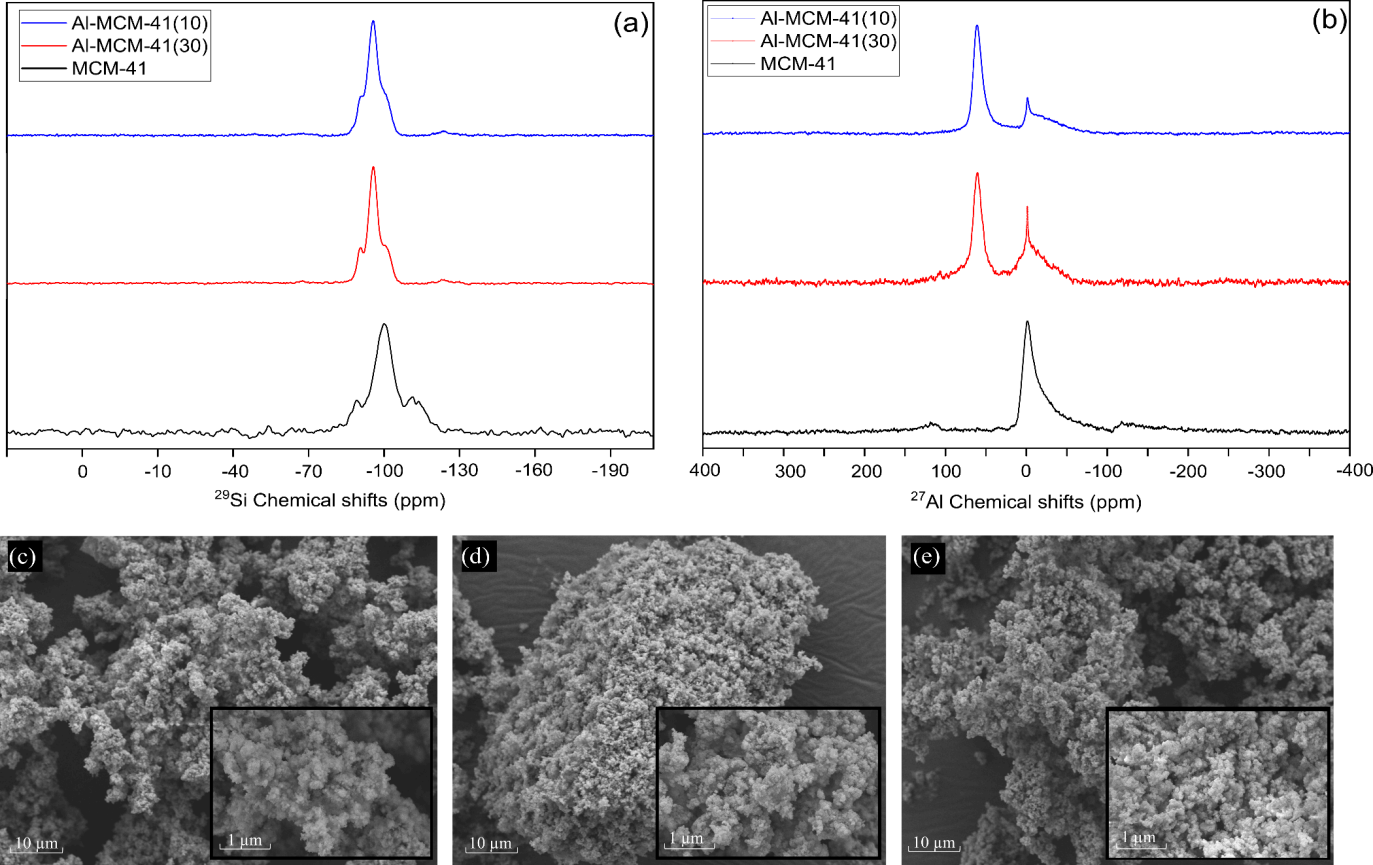
NMR spectra of (a) ^29^Si MAS and (b) ^27^Al MAS; SEM images of (c) MCM-41, (d) Al-MCM-41(30), and (e) Al-MCM-41(10).

In Figure 3a, the ^29^Si spectra show three strong signals at – 110, – 100, and – 90 ppm, mainly of the MCM-41 material. This is due to the Q^4^ [Si­(OSi)_4_], Q^3^ [Si­(OH)­(OSi)_3_], and Q^2^ [Si­(OSi)_2_(OH)_2_] Si nucleus. After Al incorporation, the three signals decreased, prevailing over the signal of −100 ppm, suggesting that the samples presented a comparable distribution of silicon sites within the mesoporous framework. Similar behavior was observed in the studies of Celoria et al.^42^ The ^27^Al MAS NMR results are shown in Figure 3b. For MCM-41, only one peak referring to silicon was observed, whereas in the Al-MCM-41 samples, two peaks were observed at 55 and 0 ppm. The peak of 55 ppm can correspond to aluminum in the tetrahedral form, proving that it was incorporated into the silica wall.^43^ This higher signal and its decrease at 0 ppm in the Al-MCM-41(10) sample show that silicon was replaced by aluminum as it increases the Al percentage in the synthesis.

SEM images reveal irregular and slightly rough crystals, mainly for the MCM-41 and Al-MCM-41(30) materials (Figure 3c,d), as observed by Chen et al.^44^ The Al-MCM-41(10) sample (Figure 3e), on the other hand, presents more spherical contours, similar to what was found by Yang et al.^45^ and Lin et al.^16^ Notably, the material morphology remains unchanged even after the Al incorporation into the MCM-41 support structure.

### Adsorption Equilibrium Studies

The single adsorption of siloxanes at 25 °C on the adsorbents MCM-41, Al-MCM-41(30), and Al-MCM-41(10) was carried out. Isotherms were calculated by using the Langmuir, Dubinin–Radushkevich, and Temkin models. The adsorption isotherms for the single (L5, D4, D5, and D6) equilibrium adsorption can be seen in Figure 4. The isotherm constants and correlation coefficients for the single-component adsorption of the siloxanes under study are listed in Table 2.

**Figure 4:**
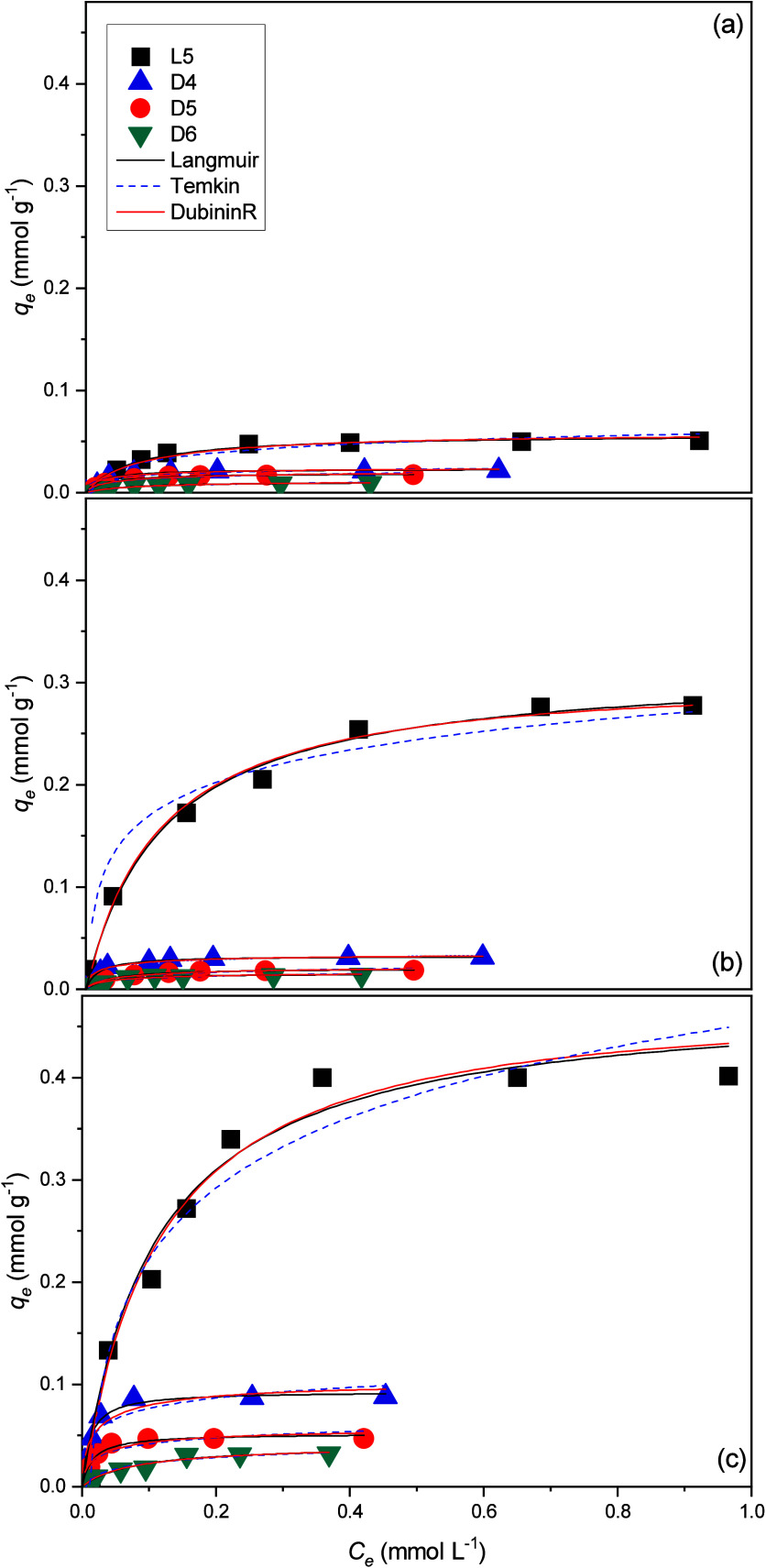
Adsorption isotherms of single-component solution (L5, D4, D5, and D6) at 25 °C on (a) MCM-41, (b) Al-MCM-41(30), and (c) Al-MCM-41(10).

**Table 2:** Equilibrium Parameters of Single-Component Adsorption (L5, D4, D5, and D6) at 25 °C on MCM-41, Al-MCM-41(30), and Al-MCM-41(10)

		Langmuir			Dubinin–Radushkevich			Temkin		
adsorbent	adsorbate	*q*_max_ (mmol g^–1^)	*K*_L_ (L mmol^–1^)	*R* ^2^	*q*_m_ (mmol g^–1^)	*E* (kJ mol^–1^)	*R* ^2^	*A*_T_ (L g^–1^)	*B* (J mol^–1^)	*R* ^2^
MCM-41	L5	0.058	13.9	0.962	0.057	4.05	0.954	0.220	5.92	0.927
	D4	0.023	47.7	0.962	0.024	4.82	0.931	4.570	1.34	0.901
	D5	0.019	27.2	0.992	0.021	4.65	0.976	1.170	1.25	0.956
	D6	0.011	16.6	0.945	0.011	4.52	0.932	0.296	1.11	0.928
Al-MCM-41(30)	L5	0.342	5.73	0.988	0.309	6.92	0.986	0.275	26.2	0.915
	D4	0.033	55.0	0.996	0.034	4.96	0.988	5.620	1.93	0.969
	D5	0.021	27.2	0.996	0.021	4.56	0.985	1.501	1.68	0.968
	D6	0.015	34.1	0.974	0.016	4.43	0.952	1.051	1.28	0.933
Al-MCM-41(10)	L5	0.479	9.13	0.960	0.462	7.07	0.963	0.437	40.1	0.961
	D4	0.093	85.8	0.992	0.100	6.80	0.957	6.571	5.47	0.938
	D5	0.052	62.9	0.978	0.056	5.97	0.933	2.991	3.85	0.908
	D6	0.040	12.9	0.964	0.040	5.72	0.967	2.570	3.57	0.953

The values of the adsorption capacity according to the Langmuir isotherm for the L5, D4, D5, and D6 molecules were, respectively, 0.058, 0.023, 0.019, and 0.011 mmol g^–1^ on MCM-41; 0.342, 0.033, 0.021, and 0.015 mmol g^–1^ on Al-MCM-41(30); and 0.479, 0.093, 0.052, and 0.040 mmol g^–1^ on Al-MCM-41(10). The maximum values of *q*_max_ estimated by the Langmuir and Dubinin–Radushkevich models are consistent with the experimentally obtained values. The best fit observed in the Langmuir isotherm (*R* ^2^ > 0.960) for all adsorbents and adsorbates indicates a predominantly monolayer adsorption. The adsorption energy calculated using the Dubinin–Radushkevich model was below 8 kJ mol^–1^ in all cases, indicating that the adsorption process was predominantly driven by physisorption.^46^ The Temkin isotherm also exhibited a good correlation with the experimental data, with *B* values showing a direct proportionality to *q*_max_ across all adsorbent systems (L5 > D4 > D5 > D6). This trend suggests that higher adsorption capacities are associated with increased sorption heat, especially for the Al-MCM-41(10) material, which presents the greatest adsorption capacity of all siloxanes under study. This behavior can also be justified by the results obtained in the characterizations. Despite a modest reduction in pore volume relative to MCM-41 (0.701 to 0.601 cm^3^ g^–1^), the Al incorporation onto the structure determined considerable increases in acidity (149 μmol NH_3_ g^–1^), pore diameter (2.96 nm), and BET surface area (812 m^2^ g^–1^). This directly influenced the better adsorption of all siloxanes, considering that MCM-41, which already has good intrinsic properties, was further improved with the incorporation. Similar behavior was also observed by Soliman et al. after MCM-41 functionalization.^47^

As observed in the single-component study, the linear compound exhibits significantly different adsorption behavior compared with the cyclic siloxanes, characterized by substantially higher *q*_max_ values. Despite the higher Al-MCM-41(10) surface area and acidity, the decrease in pore diameter (2.96 nm) may have hindered the diffusion of the more voluminous cyclic siloxanes on the material surface. A study by Balogun et al.^48^ observed that diffusivity decreases with the size of the molecules that diffuse between the cyclic siloxanes, considering that the kinetic diameters of D4, D5, and D6 are 0.98, 1.18, and 1.19 nm, respectively. These properties do not appear to influence the adsorption of a linear siloxane such as L5, since other properties, such as the acidity of the siloxane and the adsorbent and the chain opening, play a more significant role in improving accessibility and facilitating interaction with the active sites of the modified MCM-41.

Therefore, in this stage of the work, the competitive adsorption of L5 siloxane with the cyclic siloxane of the most similar molar mass (D5) and containing the same number of silicon atoms in the organic structure was evaluated. Furthermore, a ternary-component adsorption study was carried out to evaluate the effect of chain length and structural polarity (−Si–O)_*n*_ on the adsorption of cyclic compounds. Both multicomponent adsorption studies were performed at room temperature in isooctane solution under the same operating conditions as the single-component adsorption; however, only the most efficient adsorbent was evaluated. The adsorption isotherms for the binary (L5 and D5) and ternary (D4, D5, and D6) equilibrium adsorption of cyclic siloxanes onto Al-MCM-41(10) can be seen in Figure 5. The isotherm constants and correlation coefficients (*R* ^2^) for the multicomponent adsorption of siloxanes under study are presented in Table 3.

**Figure 5:**
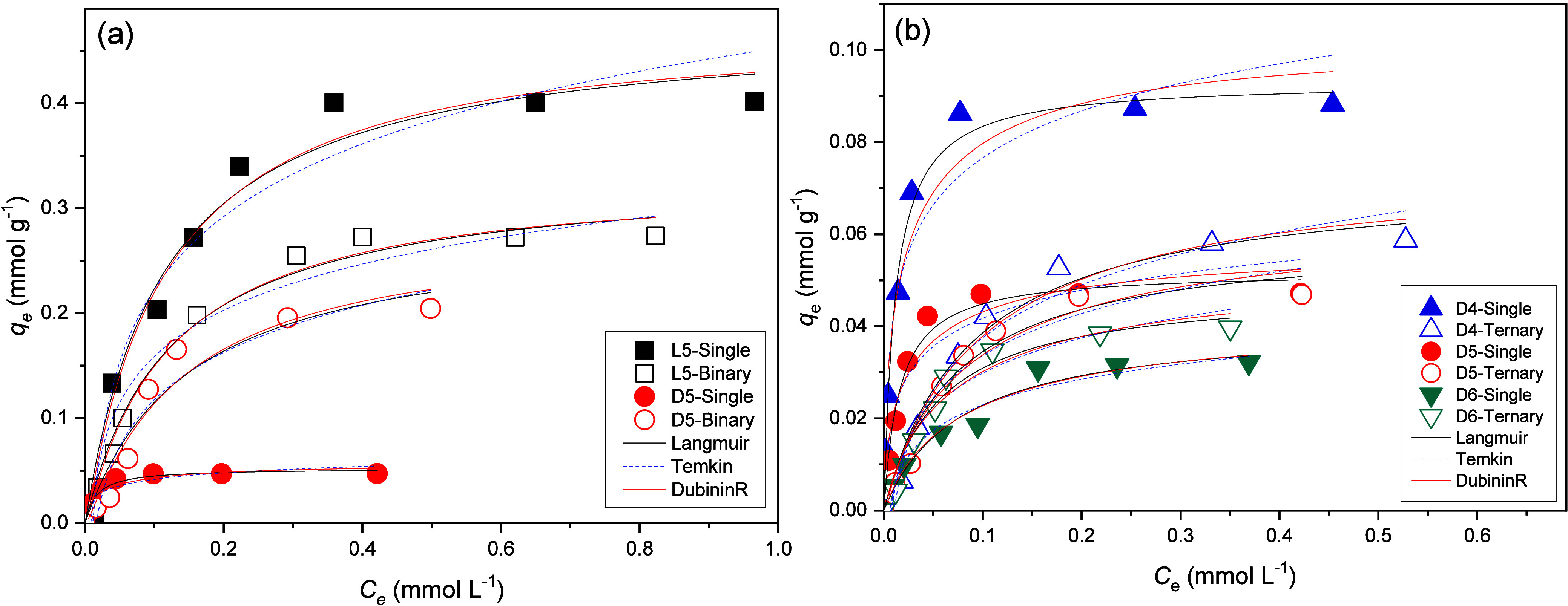
Adsorption isotherms at 25 °C on Al-MCM-41(10) of (a) L5 and D5 (single and binary components) and (b) D4, D5, and D6 (single and ternary components).

**Table 3:** Equilibrium Parameters in Adsorption of Single, Binary, and Ternary Components at 25 °C on Al-MCM-41(10)

	Langmuir			Dubinin–Radushkevich			Temkin		
adsorbate	*q*_max_ (mmol g^–1^)	*K*_L_ (L mmol^–1^)	*R* ^*2*^	*q*_m_ (mmol g^–1^)	*E* (kJ mol^–1^)	*R* ^*2*^	*A*_T_ (L g^–1^)	*B* (J mol^–1^)	*R* ^*2*^
**L5-single**	0.479	9.13	0.960	0.462	7.07	0.963	0.437	40.1	0.961
L5-binary	0.334	8.20	0.987	0.316	5.00	0.989	0.311	24.5	0.955
D4-single	0.093	85.8	0.992	0.100	6.80	0.957	6.571	5.47	0.938
**D4-ternary**	0.072	11.3	0.975	0.072	4.90	0.973	0.243	6.57	0.965
**D5-single**	0.052	62.9	0.978	0.056	5.97	0.933	2.991	3.85	0.908
**D5-binary**	0.285	6.79	0.924	0.266	4.22	0.935	0.166	24.0	0.928
**D5-ternary**	0.059	13.7	0.962	0.060	4.56	0.954	0.222	4.97	0.946
**D6-single**	0.040	12.9	0.964	0.040	5.72	0.967	2.570	3.57	0.953
**D6-ternary**	0.048	17.5	0.967	0.050	4.34	0.955	0.277	4.75	0.959

The equilibrium parameters in the binary and ternary studies presented the best fit to the Langmuir isotherm (*R* ^2^ between 0.924 and 0.987), with the Dubinin–Radushkevich *q*_max_ close to those estimated in the Langmuir model. The free energies of adsorption (*E*) confirmed the physical adsorption mechanism (E < 8 kJ mol^–1^) as verified in the single modes. Regarding the Temkin constants in the ternary experiment, an increase was observed for all molecules (D4, D5, and D6) on Al-MCM-41(10), reinforcing the competition behavior also verified in the binary mode between L5 and D5. A slight competition can be observed for D5 and D6 adsorption on Al-MCM-41(10). The slight increase in the adsorption capacities (*q*_max_) in this case confirms the greater surface activity for adsorption on aluminosilicates with appreciable amounts of acid sites (Brönsted and Lewis) about silica (MCM-41), which is weakly acidic or nearly neutral.

### Adsorption Kinetic Studies

The kinetic studies were performed at room temperature using the most effective adsorbent, evaluating the siloxanes L5 and D5 in single and binary modes and the cyclic siloxanes D4, D5, and D6 in single and ternary modes. The kinetic profiles based on pseudo-first-order, pseudo-second-order, and intraparticle diffusion models are presented in Figure 6, while the corresponding kinetic parameters for Al-MCM-41(10) across the three models are summarized in Table 4.

**Figure 6:**
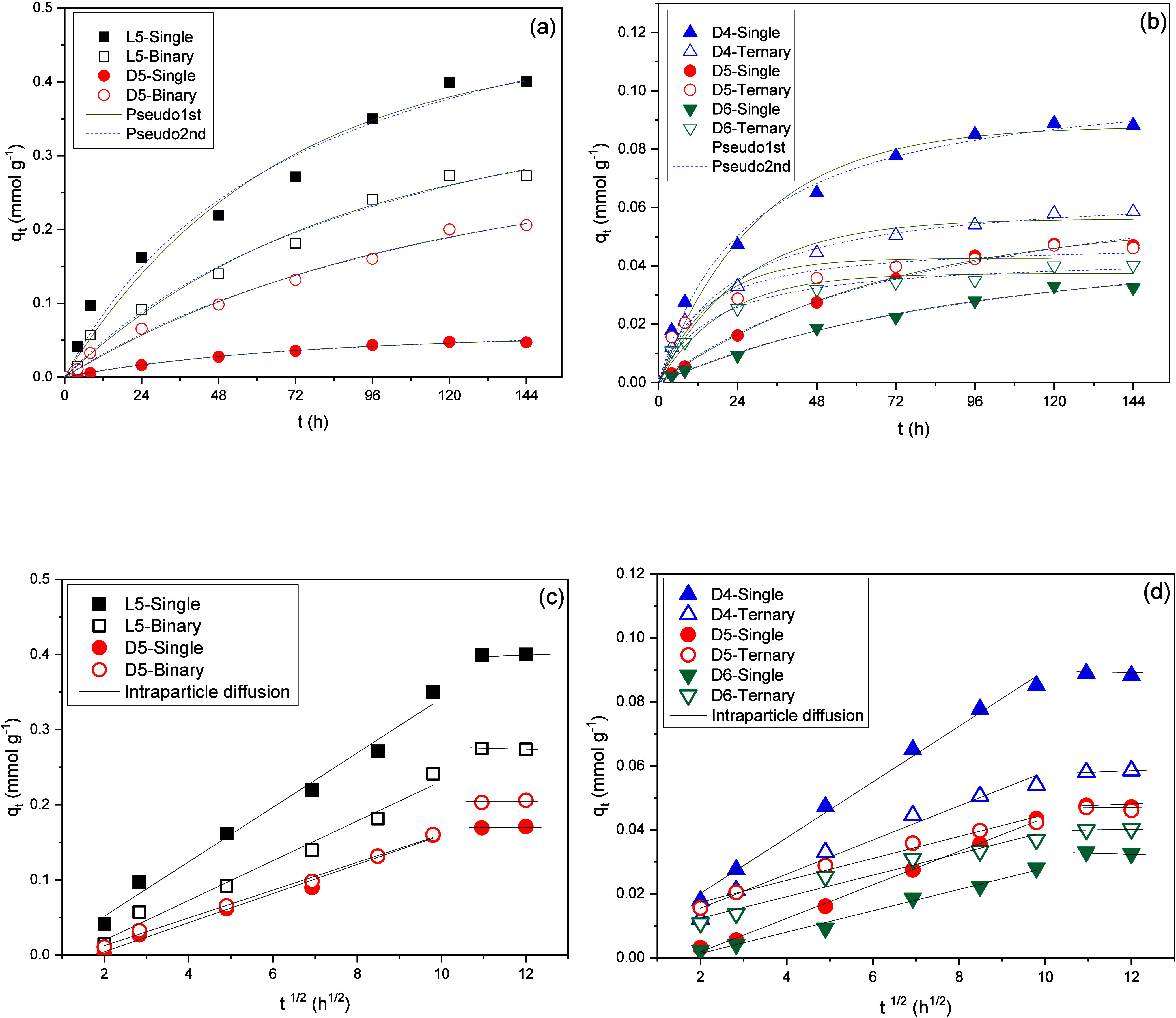
Adsorption kinetics on Al-MCM-41(10) at 25 °C of (a) L5 and D5 siloxanes (single and binary) in pseudo-first- and -second-order models, (b) D4, D5, and D6 (single and ternary) in pseudo-first- and -second-order models, (c) L5 and D5 siloxanes (single and binary) in the intraparticle diffusion model, and (d) D4, D5, and D6 (single and ternary) in the intraparticle diffusion model.

**Table 4:** Kinetics Parameters for the Adsorption of L5 and D5 (Single and Binary) and D4, D5, and D6 (Single and Ternary) at 25 °C on Al-MCM-41(10)

adsorbate(*C*_0_ = 0.50 mmol L^–1^)		pseudo-first order			pseudo-second order			intraparticle diffusion	
system	*q*_e exp_ (mmol g^–1^)	*q*_*t*_ (mmol g^–1^)	*k*_1_ (h^–1^)	*R* ^2^	*q*_*t*_ (mmol g^–1^)	*k*_2_ (g mmol^–1^ h^–1^)	*R* ^2^	*k*_int_ (mmol g^–1^ h^1/2^)	*R* ^2^
**L5-single**	0.401	0.448	0.050	0.973	0.610	0.295	0.979	0.0364	0.987
D4-single	0.088	0.088	0.033	0.983	0.106	0.187	0.993	0.0069	0.941
D5-single	0.047	0.056	0.014	0.996	0.081	0.133	0.995	0.0045	0.982
**D6-single**	0.032	0.041	0.011	0.993	0.062	0.126	0.992	0.0031	0.979
**L5-binary**	0.273	0.345	0.021	0.984	0.504	0.177	0.984	0.0266	0.984
**D5-binary**	0.203	0.280	0.019	0.990	0.429	0.088	0.991	0.0185	0.980
**D4-ternary**	0.059	0.057	0.075	0.969	0.078	0.067	0.984	0.0050	0.966
**D5-ternary**	0.046	0.043	0.063	0.927	0.049	0.047	0.965	0.0036	0.944
**D6-ternary**	0.040	0.035	0.042	0.936	0.046	0.023	0.975	0.0033	0.956

Figures 6a,b shows that the siloxane adsorbed amount increases with contact time. The kinetic profiles in all scenarios suggest that single, binary, and ternary siloxane adsorption follows pseudo-first-order kinetics. The *R* ^2^ for this model ranged from 0.927 to 0.996 (Table 4). The second-order model also shows high *R* ^2^ values (≥0.965); however, the estimates of *q*_*t*_ values are very different from capacities obtained experimentally (*q*_e exp_). As can be seen, the cyclic siloxanes reach equilibrium more quickly when compared to the linear compound (L5), which reaches equilibrium between 120 and 144 h. The equilibrium times for siloxanes D4, D5, and D6 are 72 to 96 h in the single mode and 24 to 48 h in the ternary mode. Particularly in the binary scenario on the Al-MCM-41(10) material, a strong tendency for competition between L5 and D5 is observed at the beginning of the experiment for the first 8 h of interaction, which determines very close rates, different from what occurs in the single mode. In the ternary system, this competition for access to the pore surface is marked for the first hours of contact (2 to 10 h), determining a rapid occupation of the adsorbent surface.

The linear graph of *q*_*t*_ vs *t* ^1/2^ derived from the intraparticle diffusion model (Figure 6c,d) characterizes the internal mass transfer mechanism, where the intersection point on the *y*-axis corresponds to the boundary layer thickness. The fact that the lines are not perfectly straight but have multiple slopes means that more than one step happens in the adsorption process.^26,46^ The absence of the initial rapid phase, generally linked to external surface adsorption, indicates that the adsorption mechanism initiates directly with intraparticle diffusion, which is the rate-limiting step. This diffusion phase continues for approximately 4 h and is complete after 100 h. In the last step, which corresponds to system equilibrium, the intraparticle diffusion rate decreases due to the reduced adsorbate concentration in the solution. In the binary experiment, stronger adsorptive competition between L5 and D5 was observed between 4 and 9 h, whereas in the ternary system, competition among D4, D5, and D6 was more prolonged, occurring from 4 to 25 h. This distinct behavior can be seen in Table 4 through the *k*_int_ values that presented a variation profile similar to those observed for the adsorption capacities (*q*_max_), the free energy of adsorption (*E*), pseudo-first-order constant (k^–1^), as described below: L5 ≫ D4 > D5 > D6 (single), L5 > D5 (binary), and D4 > D5 ≥ D6 (ternary).

### Regenerability Studies

The reuse profiles for the adsorptive system D4/Al-MCM-41(10) with the highest adsorption capacity among the cyclic siloxanes under study (*q*_*e*_ = 0.089 mmol L^–1^, 26.4 mg g^–1^) after successive adsorption cycles were performed. Figure 7 shows the recovery study.

**Figure 7:**
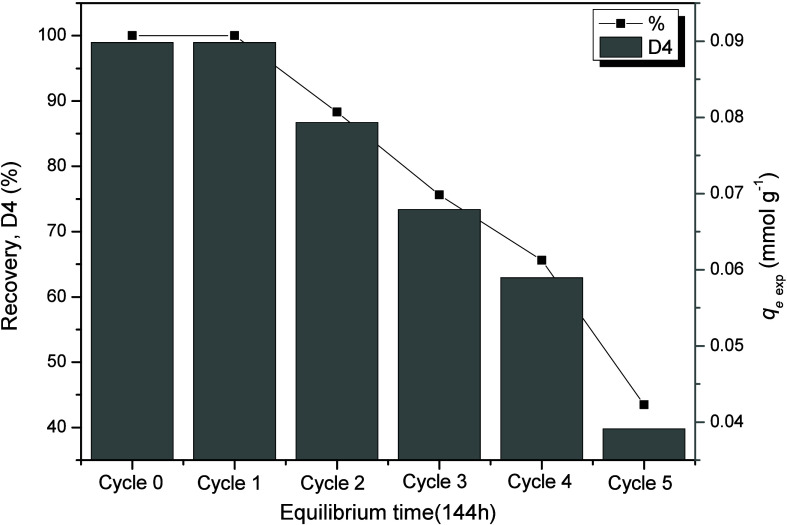
Consecutive adsorption cycles of D4 (0.67 mmol L^–1^, 200 mg L^–1^) on the Al-MCM-41(10) material after 144 h of contact.

The Al-MCM-41(10) presented a 100% adsorptive capacity for two complete use tests, considering the observed *q*_max_ values. From the second adsorption cycle onward, the adsorption capacity was approximately 88%, and in the third adsorption cycle, it remained high, for a moderate removal of 76%, approximately. In the fourth and fifth reuse cycles, the adsorptive capacities were 66 and 44%, which can already be considered unproductive from a technical or commercial perspective.

The reduction in compound adsorption over successive reuse steps can be explained by the decrease in active surface groups (Si–OH, Al–OH, etc.) caused by dehydration/restructuring during repeated calcinations.^49^ The MCM-41 mesoporous structure can be damaged when manipulated at high temperatures, causing a decrease in the specific surface area and pore volume. This structure can be preserved only when the mesoporous silica still contains the modeling surfactant inside the pores. When the surfactant is completely evaporated and subjected to consecutive calcination processes, the structure may collapse.^50^ Despite this, some studies have shown that incorporating Al into the silica structure improves hydrothermal stability.^51^ Therefore, the effect of adsorption capacity loss was only noticed to a greater degree from the fourth reuse cycle onward.

## Discussion

Some effects can be considered determinants for the adsorption order, as they involve the adsorbate and adsorbent nature. The siloxane effects are influenced by the geometry, polarity, and acidity, and the adsorbent effects are determined by polarity and material acidity (related to aluminum incorporation into the composition/structure).

The effect of the siloxane geometry can be observed from the adsorption characteristics of the linear compound and the other cyclic siloxanes in the study. In a single-component solution, the L5 adsorption capacities on all of the adsorbents, MCM-41, Al-MCM-41(30), and Al-MCM-41(10), are approximately 2.5, 10.4, and 5.2 times higher than those observed for D4 (the most strongly adsorbed cyclic siloxane). This behavior can be attributed to the molecular linearity since the open chain provides greater electronic availability and a larger contact area for adsorption on the surface of the adsorbent, which does not occur with compounds with a cyclic structure, which generally have greater steric hindrances. This can be confirmed through the previous study by Alves et al.,^22^ where DFT simulations corroborated the experimental results with MCM-41, showing an energy of −0.76 eV for the MCM-L5 system, compared to −0.50 eV in MCM-D5. Applying this to the present study, the low energy required by the adsorbents to interact with the linear pentasiloxane indicates a greater ease of adsorption, indicating stronger van der Waals forces between the −OH functional groups of the siloxane and the surface-exposed oxygen atoms of MCM-41 and Al-MCM-41. The adsorbent structures in the study consist of Si–O–Si and Si–O–Al bonds that can act as active sites in the adsorption process, leading to a network of Si–O–Si bonds in the adsorptive systems.^52^

In contrast to linear siloxanes, the molecular stability observed in higher-molecular-weight cyclic siloxanes can be associated with a greater number of O–Si–O bonds. These structural units likely demand more energy to distort the oxygen atoms from their tetrahedral geometry, particularly due to the reduced presence of methyl groups surrounding the cyclic framework.^5^ A study by Tran et al.^53^ with activated carbons (AC) indicates that, for linear siloxanes, the adsorption capacity rises with increasing chain length, while an opposite trend is found for cyclic species. The polarity effect of the cyclic molecule can be observed in the estimates of the adsorption capacities, particularly when comparing cyclic siloxanes. In general, cyclic molecules have greater stability compared to linear ones. There is a tendency for cyclic compounds with lower molecular weight to be more intensely adsorbed, as can be seen in the *q*_max_ results for all the adsorbents investigated, which led to an adsorption order of D4 > D5 > D6 for the solids MCM-41 and Al-MCM-41(30) and D4 ≫ D5 > D6 for Al-MCM-41(10).

Concerning siloxane acidity effects, their amphiphilic structural properties are related to the presence of the polar inorganic structure (Si–O bond) and nonpolar organic functional groups (−CH_3_), which may be determinants for this behavior.^54,55^ When the polar and nonpolar effects of the siloxane structure are compared, it is seen that the electron pairs around oxygen are not easily available for electron donation. In this context, the ligand deformation energy after complexation plays an important role. A study by Passmore et al.^56^ showed in an energy decomposition analysis comparing 1,3-dimethylsiloxane and diethyl ether that siloxane was more difficult to complex due to the high polarization of silicon atoms, resulting from repulsive interactions (M^+^··· Si^δ+^). In general, siloxane requires a higher energy expenditure related to its structural and conformational changes to achieve the same level of interaction with electrophiles. In this domain of interactions, Cameron et al.^57^ found a weaker electrostatic attraction of D6 to M^+^ (M = Li, Ag), when compared to the 18-crown-6 ether. Mojsiewicz-Pieńkowska et al.^54^ reported that the flexibility caused by the interconversion of configurations is a characteristic that allows the arrangement of methyl groups on the surface, with a consequent reduction in surface tension and a decrease in surface energy. Thus, siloxane D6 with 12 structural methyl radicals has a greater tendency to lower surface energies when compared with D5 and D4 molecules with 10 and 8 methyl groups, respectively. This behavior may be related to the lower D6 adsorption capacity with all of the adsorbents under study. In addition, the larger size and molecular volume of D6 determine a greater impediment (in addition to the greater electronic repulsion) in the access of large molecules to the adsorptive sites on the surface of the adsorbents, especially at higher concentrations.

The present study observed an increasing adsorption trend in the D6 < D5 < D4 order for cyclic siloxanes. Tran et al.^53^ reported gas-phase-related *q*_max_ values for cyclic siloxanes on activated carbon with decreasing adsorption of 161 and 146 mg g^–1^ (0.543 and 0.394 mmol g^–1^) for D4 and D5, respectively. A study by Silva et al.^20^ in the liquid phase (*n*-octane) using different silicon adsorbents (>96% SiO_2_, 1–5 nm) revealed maximum adsorption capacities of D4 of 116 mg g^–1^ (0.391 mmol g^–1^) on white silica gel (WSG), 32 mg g^–1^ (0.108 mmol g^–1^) on blue silica gel (BSG), 72 mg g^–1^ (0.243 mmol g^–1^) on silica derived from waste (SS), and 178 mg g^–1^ (0.480 mmol g^–1^), 59 mg g^–1^ (0.159 mmol g^–1^), and 16 mg g^–1^ (0.043 mmol g^–1^) for the adsorption of D5 on WSG, BSG, and SS, respectively. Another study used hierarchical CFAU composites with Ag^+^ or Cu^2+^ to test the removal of small linear siloxanes (MMST, TMS, and DMSD) and DMSO_2_ from water at room temperature and neutral pH. Single- and multicomponent adsorption data showed that the incorporation of Ag^+^ significantly improved the adsorption capacity, particularly for single-component TMS, DMSD, and DMSO_2_ (with adsorption capacities of 1.05, 0.72, and 15.75 mg g^–1^, respectively).^52^

In the adsorbent acidic nature effect, a notable increase in adsorption was observed with the increase in the Al molar proportion onto the MCM-41 matrix, with higher *q*_max_ values for all siloxanes observed on the Al-MCM-41(10) material, where moderate-strength Lewis acid sites predominate. The low *q*_max_ values for L5 (0.058 mmol L^–1^), D4 (0.023 mmol L^–1^), D5 (0.019 mmol L^–1^), and D6 (0.011 mmol L^–1^) on the MCM-41 material are attributed to diffusion limitations, low accessibility, and the absence of acid sites inside the mesoporous materials, which hinders the formation of the adsorptive complex between the ligand (molecule) and the receptor (adsorbent solid). Conversely, mesoporous aluminosilicates with larger surface areas and significantly higher acidity exhibit greater surface interaction with siloxane molecules, leading to lower energies for the formation of adsorbed complexes. In this context, discussions on the covalent and ionic characteristics of siloxanes, associated with the oxygen basicity atoms and the Si–O bonds, should be considered. Dankert and von Hanisch (2021)^1^ reported that Si–O groups can be good electron donors at small bond angles, such that the basicity of siloxanes increases the smaller the Si–O–Si angle, determining that both covalence and ionicity increase simultaneously in interactions where larger Si–O–Si angles are required. Thus, D4 with tetrahedral geometry is more apolar than D5 and D6, consequently more basic, with a smaller molecular volume and less sterically hindered, and is more easily attracted to the aluminosilicate surface through interactions with Lewis acid centers. It is inferred that the Al-MCM-41(10) surface is more susceptible to accepting a pair of electrons from the Si–O groups and, thus, forming a more stable coordinated covalent bond with a Lewis base.

This behavior is more easily observed when comparing the adsorption of cyclic siloxanes. In this case, the high adsorption capacity in the order D4 > D5 > D6 results from the greater energetic interactions of the MCM-41/siloxane ligand complexes. Furthermore, due to its amphiphilic nature, siloxane can form hydrogen bonds from oxygen atoms (Si–O), which can determine adsorption forces on more acidic materials containing Si or Al groups, responsible for Brönsted acidity on the surface.^54^ Cabrera-Codony et al.^58^ evaluated the use of three distinct categories of carbonaceous materials containing micropores and micro-/mesopores or activated with phosphoric acid for L2, D4, and D5 siloxanes in the gas phase. The activated carbons with an acidic character and a mesoporous structure had the highest adsorption capacity compared with less acidic or microporous adsorbents. The adsorption capacities varied in 900–1400, 350–550, and 200–250 mg g^–1^ ranges for D5, D4, and L2, respectively. Zhong et al.^59^ synthesized mesoporous aluminas with uniform pores for the adsorption of D4. The study showed that nanostructured silica and commercial alumina have Lewis acid sites, with the synthesized sample containing major acidity. The results showed that the material with the greater volume of mesopores reached a *q*_max_ of 168 mg g^–1^ (0.567 mmol L^–1^) in the siloxane adsorption. This value was approximately 30% higher than that of commercial alumina.

Concerning the binary study, it is notable that there is an adsorptive competition effect when the siloxanes interact on the surface and pore network of the aluminosilicate, where D5 *q*_max_ increases approximately 5.5 times to the detriment of the reduction in the adsorptive capacity of L5. Similar behavior was observed in the study of this research group using only the pure MCM-41 matrix in D5- and L5-binary studies, where a decrease in L5 and an increase in D5 (2 times) were also observed.^22^ The effects of greater polarity in the cyclic molecule, combined with the greater surface acidity resulting from Al incorporation into the structure, compensate for the lower hindrance of the linear species and may probably have important contributions to explain this behavior. A study using AC observed that acidic sites on the surface can promote the cleavage of siloxane bonds, leading to the formation of α-ω-silanediols, and then condense to form more cyclic siloxanes.^58^ This may have directly influenced the competitive adsorption through the transformation of L5 linear siloxane to cyclic siloxane, causing D5 to win the competition in the binary system. Tran et al.^53^ observed similar results using two commercial activated carbons (STIX and AP4) to investigate the adsorption capacity of different siloxanes in the gas phase. The estimated L4 adsorption capacities were 0.521 and 0.932 mmol^–1^ for STIX and AP4, respectively, while for D5, the values were 0.395 and 0.662 mmol L^–1^.

In the ternary solution, a slight competition effect can be observed for the D5 and D6 adsorptions on Al-MCM-41(10). The slight increase in the adsorption capacities confirms the greater surface activity for adsorption on aluminosilicates with appreciable numbers of acidic sites. Notably, the comparative analysis for the series of cyclic siloxanes under study, excluding the macro effect of the geometric nature, shows that adsorption decreases with the increase in the carbon chain or molecular weight of the adsorbate, these properties being more determining for the separation phenomenon than the positive effect of the number of polar groups (Si–O)_*n*_ in the structure. The adsorption order in the ternary scenario, considering the structural and textural characteristics of the synthesized materials, is D4 (296 g mol^–1^, *n* = 4) > D5 (370 g mol^–1^, *n* = 5) > D6 (444 g mol^–1^, *n* = 6). A study by Cabrera-Codony et al.^58^ also assesses the competitive adsorption in a multicomponent gas phase of limonene, toluene, and siloxanes as L2, D4, and D5, using microporous activated carbons and carbons activated with phosphoric acid containing micro and mesopores. For microporous adsorbents, the adsorption followed the order: limonene ≫ D5 > D4 > L2 ≈ toluene, while in the case of the acidic micro/mesoporous adsorbent, the order found was of the type: D5 > limonene > D4 > L2 > toluene. The acidic nature of the adsorbent, the presence of mesopores, and the increased surface functionalization with oxygen-containing groups increase the D5 adsorption capacity.

Regarding kinetic adsorption, low and slow adsorption behaviors were reported in the literature for D4 by Popat and Deshusses^60^ and for D4 and D5 by Silva et al.,^20^ corresponding to equilibrium times close to 200 h. Yang et al.^61^ reported comparable results when employing CuO-modified AC for the gas-phase D4 adsorption. The diffusion analysis revealed two well-defined stages: an initial phase governed by intraparticle diffusion, followed by a second stage corresponding to the establishment of adsorption equilibrium. In the binary and ternary mixtures, the kinetic mechanism was similar, characterized by the absence of adsorption on the internal surface and the intraparticle diffusion stage (4 to 100 h) until reaching the final equilibrium (120–144 h).

## Conclusions

Silica and aluminosilicate materials were synthesized via a room-temperature sol–gel method with high yields (84–92%), resulting in MCM-41 structures containing varying aluminum contents (0.00%, 1.3%, and 4.4 wt %). These materials exhibited excellent textural properties and effective adsorption of siloxanes, particularly Al-MCM-41(10), which showed superior performance due to its higher acidity in single-, binary-, and ternary-component systems. The adsorption capacity was strongly influenced by the siloxane linear structure, with higher capacities than cyclic ones, and D4 stood out among cyclic siloxanes due to its shorter chain, lower molar mass, and lower number of Si–O groups. Adsorption kinetics followed a pseudo-first-order model, with equilibrium reached after 144 h, and the best adsorbent maintained high adsorption capacity through three reuse cycles. In conclusion, the results of the liquid-phase siloxane adsorption studies presented in this work are expected to offer valuable technical insights for applications in both liquid and gas phases. Aluminosilicate materials with a higher aluminum content show strong potential for the treatment of surface waters and industrial effluents, particularly from cosmetics, plastics, and related sectors that contain elevated levels of siloxanes. Furthermore, their potential application in gas-phase systems, such as portable adsorbent filters for siloxane removal in gas pipelines, may contribute to the development of new methodologies for energy performance improvement of biogas or biomethane.

## Supplementary Material



## Notes

Gels molar compositions with a respective Si/Al ratio and XRD structural parameters of the synthesized materials (PDF)

## #

Materiales Funcionales para Tecnologías del Hidrógeno (FUNMATECH2), Centro de Investigaciones Energéticas, Medioambientales y Tecnológicas, Av. Complutense, 40, 28040- Madrid, Spain
