# Supplementary material for: Single- and Multicomponent Siloxane Adsorption in Al-MCM-41 (Al = 0.0, 1.3, and 4.4)
Source: ACS Omega. 2025 Sep 8;10(36):41014–28. doi: 10.1021/acsomega.5c03379 (PMC12444551; doi:10.1021/acsomega.5c03379)
Supplement: Supplementary file 1 [file ao5c03379_si_001.pdf]

Support Information:

Single- and multi-component siloxanes adsorption in Al-MCM-41 (Al = 0.0, 1.3 and 4.4)

Camila M. A. C. Alves [a,*,†], *Júlia F. Alves* [b], *Raimundo C. Rabelo-Neto* [c], *Luiz S. C. Júnior* [d], *Araceli Fuerte* [e], *Paloma Ferreira-Aparicio* [e], *Rita X. Valenzuela* [e], *Rinaldo S. Araújo* [b], *and Mona Lisa M. Oliveira* [a]

[a] Universidade Estadual do Ceará (UECE), Fortaleza, Ceará, Brazil

[b] Instituto Federal de Educação, Ciência e Tecnologia do Ceará, Campus de Fortaleza (IFCE), Fortaleza, Ceará, Brazil

[c] Instituto Nacional de Tecnologia (INT), Rio de Janeiro, Rio de Janeiro, Brazil

[d] Centro de Pesquisas, Desenvolvimento e Inovação Leopoldo A. M. de Mello (Cenpes), Petróleo Brasileiro S.A. (PETROBRAS), Rio de Janeiro, Rio de Janeiro, Brazil

[e] Centro de Investigaciones Energéticas, Medioambientales y Tecnológicas (CIEMAT), Madrid, Spain.

Table S1. Molar compositions of the synthesis gels and respective Si/Al ratios.

**Sample**	**Gels composition molar**	**Molar ratio Si/Al**
**MCM-41**	1 TEOS: 0.30 dodecylamine: 5.2 EtOH: 25 H_2O	-
**AL-MCM-41(30)**	1 TEOS: 0.033 AlTIPO: 0.30 dodecylamine: 5.2 EtOH: 25 H_2O	10
**AL-MCM-41(10)**	1 TEOS: 0.10 AlTIPO: 0.30 dodecylamine: 5.2 EtOH: 25 H_2O	30

Table S2. Structural parameters by XRD of the synthesized materials.

**Adsorbent**	**d_{100} / nm**	**a_0 / nm**
**MCM-41**	3.86	4.45
**AL-MCM-41(30)**	3.87	4.47
**AL-MCM-41(10)**	3.85	4.45
